# Novel Colloidal Dispersing Concept in Aqueous Media for Preparation by Wet-Jet Milling Dispersing Method

**DOI:** 10.3390/nano13010080

**Published:** 2022-12-24

**Authors:** Haruhisa Kato, Ayako Nakamura

**Affiliations:** National Metrology Institute of Japan (NMIJ), National Institute of Advanced Industrial Science and Technology (AIST), Tsukuba Central 5, 1-1-1 Higashi, Tsukuba 305-8565, Ibaraki, Japan

**Keywords:** wet-jet milling, dynamic light scattering, dispersing energy input, zeta potential, DLVO theory, calcium carbonate

## Abstract

Dispersing particles in a liquid phase is significant for producing various functional nano/bio applications. The wet-jet milling method has been gaining attention as an attractive dispersing method in the preparation of soft material suspensions. This is because the main driving force of dispersion by the wet-jet milling method is the shear force, which is weaker than that it is in the ultrasonication dispersing method. In the wet-jet milling method, the pressure of the narrow channel which the liquid is passes through and the number of passes are used as the control parameters for dispersing the particles. However, the values of the pressure depend on the size (diameter and length) of the narrow channel, thus, it is not a commonly used dispersing parameter in dispersing by wet-jet milling to set the dispersing condition by various wet-jet milling instruments. In addition, wet-jet milling users must optimize the dispersing conditions such as the pressure and number of passes in the narrow channel, therefore, a simple prediction/optimization method of the dispersing size by the wet-jet milling method is desired. In this study, we established a novel colloidal dispersing concept, the dispersing energy input based on a calorimetric idea, for particle suspension preparation using the wet-jet milling method. The dispersing energy input by wet-jet milling was quantitatively calculated under various conditions during the dispersing by wet-jet milling, and then, the dispersing size of the particles was easily predicted/optimized. We demonstrated the usability of the concept by preparing aqueous suspensions of calcium carbonate (CaCO_3_) particles with various surfactants using the wet-jet milling method. Based on the established concept, in a case study on dispersing CaCO_3_, we found that changes in the micelle sizes of the surfactants played a role in wet-jet milling. The novel idea of the representation of energy input makes it possible to estimate the appropriate condition of the dispersing process by wet-jet milling to control the size of particles.

## 1. Introduction

There are various material dispersion applications, such as in paint preparation, cosmetics, drug delivery systems, photo resists, detergents, coating materials, glues, and food processing. For the preparation of these applications, suspending various materials in liquid media is a crucial processing stage because this process is the first step in the manufacturing stage [1,2,3,4,5]. There are various methods for suspending materials in the liquid phase, such as vortex mixing, jet milling [6,7,8], and using an ultrasonic bath [9,10] or an ultrasonic homogenizer [11,12]. The wet-jet milling method has been recently utilized as an attractive dispersing method in preparing soft material suspensions because it involves a weaker destructive force compared with that in the ultrasonication dispersing method.

The wet-jet milling method is a wet-type milling method that is used to disintegrate agglomerates of powder/polymer samples in a liquid phase. In this method, which is also called high-pressure homogenization, the particles dispersed in a liquid are passed through a narrowed channel at a high pressure. Subsequently, the dispersion of particles is enhanced by the complexed shear force arising from the turbulent flow in the channel. The advantage of this dispersing technique is that it yields suspensions with low contamination, unlike the ultrasonic homogenizer method, inducing the contamination of the sonication tip. Weak destructive forces, such as the shear force, are also attractive to disperse soft materials in a liquid phase, therefore, the wet-jet milling method has been used to disperse carbon nanotubes, cellulose, and polymeric materials in a liquid phase. This method is fundamentally utilized in food processing because the shear force does not induce the denaturization of food materials. This method has also been used to disperse hard materials, such as graphene and metal oxide particles.

To disperse particles by the wet-jet milling method, a particle suspension which is pressurized by a pressure intensifier is accelerated by a nozzle in the narrow channel so that the dispersed particles collide with each other to achieve micronization. This operation is not enough to disperse the particles completely in one attempt, therefore, a continuous operation is necessary. The required number of repeated continuous collision of the particle suspension depends on the characteristics of the particles, such as the degree of aggregation or agglomeration. Although dispersing particles using this method requires the repeated continuous collision of the suspension through the narrow channel, it is well known that this method primarily focuses on the pressure in the narrow channel. Typically, the pressure range of a commercial wet-jet milling system is 80–245 MPa. Specifically, to describe the condition of dispersing particles in the wet-jet milling method, the pressure in the narrow channel is one of the main parameters, however, the dispersing tendency is not simply represented as a function of this pressure. Therefore, the users of wet-jet milling must optimize the pressure and change the number continuous collisions of the suspension through the narrow channel by monitoring using a simple sizing method such as dynamic light scattering (DLS) in each operation. Therefore, an easy estimation of the appropriate condition of the dispersing process using wet-jet milling to control the size of particles is desired.

Therefore, the purpose of this study was to solve the difficulty in predicting/optimizing the dispersing condition in the wet-jet milling method. In this study, we established a novel concept—the dispersing energy input—in the preparation of a particle suspension using the wet-jet milling method. The dispersing energy input was evaluated quantitatively, and we found a close relationship between the dispersing energy input and the particle size of the suspension.

In this study, we dispersed calcium carbonate (CaCO_3_) particles in aqueous suspensions with various surfactants using wet-jet milling. CaCO_3_ is currently being used in biological and industrial fields, such as in paper coating, paint, polymer molding, food, and preparation of clay materials, owing to its ease of production, high biocompatibility, and slow biodegradability [13,14,15,16,17,18,19,20,21,22]. Especially, the preparation of smaller sized CaCO_3_ nanoparticles in a liquid phase is becoming essential to produce better functional properties in various fields since reducing the size of particle induces a larger specific surface area and a higher surface energy [23,24]. Therefore, we used CaCO_3_ particles as an example in this study; predicting the appropriate dispersing condition in the wet-jet milling method to disperse CaCO_3_ particles in the required size is quite useful for their applications.

## 2. Experimental Section

### 2.1. Materials

CaCO_3_ powders (Hakuenka-CC-R) were gained from Shiraishi Kogyo Kaisha, Ltd. (Osaka, Japan). Non-pore-structured CaCO_3_ particles were used in this study. Sodium dodecyl sulfate (SDS) was bought from Wako Pure Chemical Industries, Ltd. (Osaka, Japan). Softanol-70 was gained from Nippon Shokubai Co., Ltd. (Osaka, Japan). Hexadecyltrimethylammonium bromide (CTAB) was bought from Tokyo Chemical Industry Co., Ltd. (TCI; Tokyo, Japan). Triton X-100 was bought from Thermo Fisher Scientific (Geel, Belgium).

### 2.2. Preparation of CaCO_3_ Particle Aqueous Suspensions

Homogeneous aqueous suspensions of CaCO_3_ particles were suspended in aqueous solutions of Triton X-100, SDS, Softanol-70, and CTAB using the wet-jet milling method (YUH-PA 5-14-E, Yoshida works pro., Tokyo, Japan). The final concentration of CaCO_3_ particles were 0.2 mg/mL and the concentration of all of the surfactants were 0.5 mg/mL. The pressure range of the wet-jet milling system was from 80 MPa to 240 MPa. The prepared CaCO_3_ particle dispersions were stable for at least one week.

### 2.3. DLS Measurements

A DLS particle analyzer (DLS8000, Otsuka Electronics Co., Ltd., Kyoto, Japan) was utilized. The measurements were taken using a 45 mW He–Ne laser. The observed scattering angle was 90° at 25.0 ± 0.1 °C. The measurement system was kept at a constant temperature of 23.0 ± 0.3 °C, and the humidity was controlled at 40 ± 3%. Repeated measurements were performed at least three times, and the mean values were calculated. The particle diameter was calculated by the Stokes–Einstein equation.

(1) $$d_{l}=\frac{k_{B}T}{3\pi \eta D},$$

where *k_B_* is the Boltzmann constant, *T* is the absolute temperature, *η* is the viscosity of the medium, and *d_l_* is the light scattering intensity averaged diameter of the CaCO_3_ particles in the suspensions.

### 2.4. Pulsed Field Gradient-Nuclear Magnetic Resonance Measurements

The measurements of pulsed field gradient-nuclear magnetic resonance (PFG-NMR) were taken by a 14.1 T spectrometer (UNITY INOVA 600A, Varian, California, USA) using a H-F{X} diffusion probe (DSI-V218, Doty Scientific, California, USA) capable of magnetic field pulse gradients of 2500 G cm^−1^ in the z direction. The observed temperature was at 25.0 ± 0.1 °C. The temperature calibration was completed with pure methanol. The Stejskal–Tanner diffusion equation was used for the determination of the diffusion coefficients.

(2) $$\ln(I/I0)=-D\gamma_{}^{2}G_{}^{2}\delta_{}^{2}(\Delta -\delta /3),$$

where only the data for which the correlation coefficient of ln(*I*/*I_0_*) vs. $\gamma^{2}G^{2}\delta^{2}\left(\Delta -\delta /3\right)$ was higher than 0.99 for the first decay were utilized. In this study, a PFG-stimulated echo sequence was utilized [25]. Rectangular gradients of 1 ms were gradually increased from 0 to approximately 600 G cm^−1^ at approximately 100–500 averaged transients. The other parameters are follows: the π/2 pulse width was 12.90 μs, the relaxation delay was 10 s, and the acquisition time was 2.0 s. The diffusion time (Δ) was set to 50 ms. Pure water was used to ensure the linearity of the gradient strength. A value of 2.299 × 10^−9^ m^2^ s^−1^ was utilized as the water diffusion coefficient [26].

### 2.5. Electrophoretic Mobility Measurements

The electrophoretic mobility measurements were taken using a zeta potential analyzer (Otsuka Electronics Co., Ltd., Osaka, Japan). The values were calculated using the Smoluchowski assumption. The assumption might overestimate an actual zeta potential by up to approximately 20% [27].

### 2.6. Viscosity Measurements

The viscosity of aqueous solution of each surfactant was measured using an Uberode viscometer. The observed temperature was at 25.0 ± 0.1 °C. The all of the surfactant solutions had a same viscosities of approximately 0.89 cP, which is equal to that of water (0.89 cP) [28].

## 3. Results and Discussion

### 3.1. Determination of Dispersing Energy Input by Wet-Jet Milling Method

**Figure 1:**
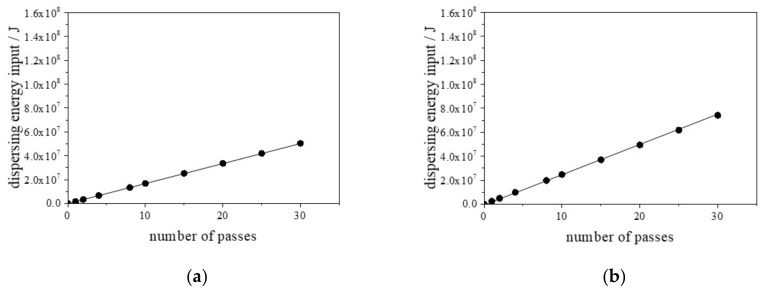

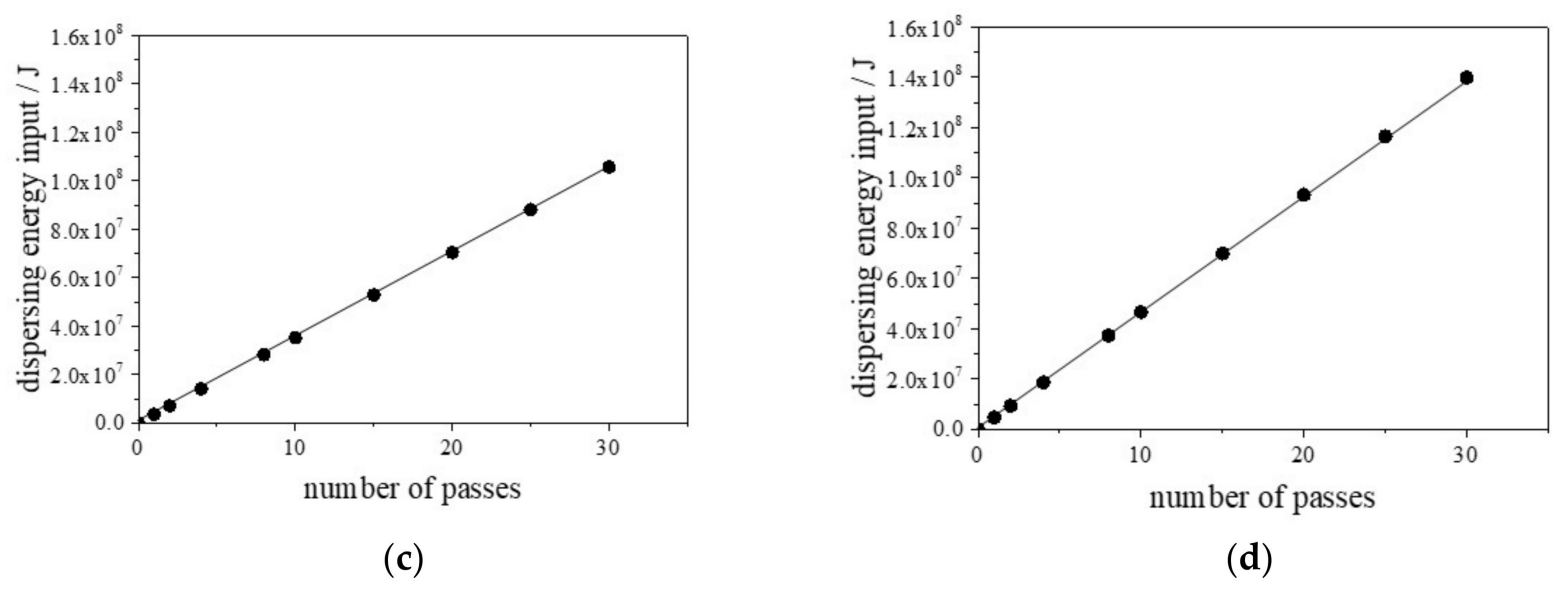
Plots of the calculated dispersing energy input as a function of the number of passes in the wet-jet milling system. The values of the dispersing energy input were evaluated at four different pressures in the narrow channel and pass time in the wet-jet milling system: (**a**) 80 MPa, (**b**) 120 MPa, (**c**) 180 MPa, and (**d**) 240 MPa, respectively.

To determine the dispersing energy input by the wet-jet milling method, we used a calorimetric idea to measure, directly, the effective energy delivered to a liquid by the wet-jet milling method. This idea is based on the measurement of the increasing temperature in a liquid by the wet-jet milling method in relation to the dispersing energy input of the liquid that is passed through the narrowed channel at a high pressure. Although the heat transfer from the material tank of a wet-jet milling system is known, in this study, we assumed the heat transfer to be zero to ensure thermal equilibration while we were passing the liquid through the channel of the wet-jet milling system. Moreover, we defined the start temperature of the water as 25 °C, following which we started wet-jet milling and recorded the temperature vs. time in the material tank of the wet-jet milling system to determine direct calorimetric curves. We evaluated the initial slope of the temperature increase after obtaining the best linear fit for each curve using least squares regression (data not shown). The delivered wet-jet milling power is expressed as:

(3) $$P_{\mathrm{cal}}=c_{p,\mathrm{water}}\times m_{\mathrm{water}}\times \frac{dT}{dt},$$

where $P_{\mathrm{cal}}$ is the delivered wet-jet milling power (W) in one pass, T and t are the temperature (K) and the passing time (s), respectively, $c_{p,\mathrm{water}}$ is the specific heat of water (4180 J/gK), and m is the mass of the liquid (one pass is 30 g in our instrument). In this study, we changed the dispersing condition by the wet-jet milling method using four different pressures (80, 120,180, and 240 MPa) in the narrow channel and pass time in the wet-jet milling system. In our wet-jet milling instrument, the observed values of $\frac{dT}{dt}$ were 3.8, 6.3, 11.6, and 17.4 K/s at the four different pressures (80, 120, 180, and 240 MPa), respectively. Therefore, using Equation (3), the evaluated values of $P_{\mathrm{cal}}$ were 4.75 × 10^6^, 7.89 × 10^6^, 1.45 × 10^7^, and 2.18 × 10^7^ W at the four different pressures (80, 120, 180, and 240 MPa), respectively. Using the evaluated $P_{\mathrm{cal}}$ at the different pressures in the narrow channel of the wet-jet milling instrument, the dispersing energy input (J) was calculated by considering the operating time (s) of one pass. Finally, the dispersing energy inputs at the four different pressures (80, 120, 180, and 240 MPa) in the narrow channels were calculated, and the results are shown in Figure 1. We used the calculated dispersing energy input as the only parameter based on the measurement of the increasing temperature in the liquid in the wet-jet milling method. The objective was to examine the relationship between the dispersing energy input and the particle size in the dispersing process of the particles in the liquid phase using wet-jet milling.

### 3.2. Particle Size Analysis of Various Aqueous CaCO_3_ Particle Dispersions

**Figure 2:**
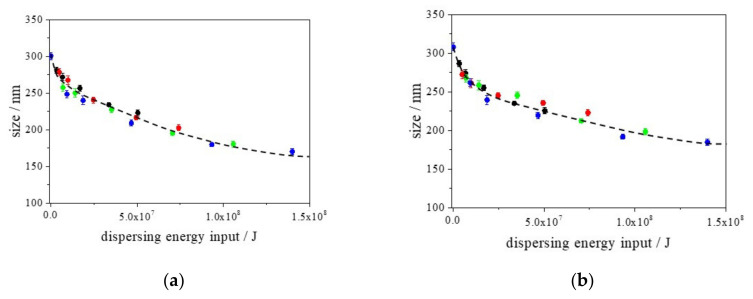

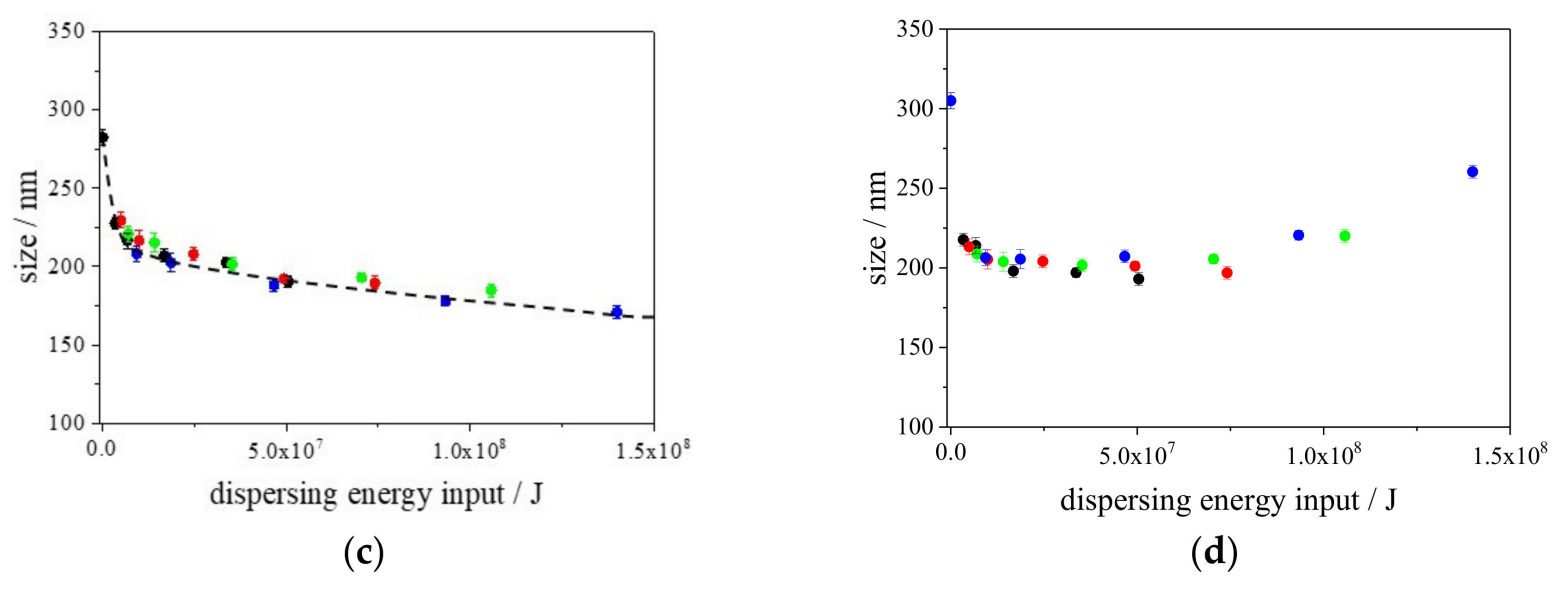
Plots of the mean sizes of CaCO_3_ particles in various aqueous media as functions of the dispersing energy input, as determined by DLS. (**a**) Triton X-100, (**b**) SDS, (**c**) Softanol-70, and (**d**) CTAB were used as surfactants. The plots are for different pressures in the narrow channel (80 MPa: black, 120 MPa: red, 180 MPa: green, and 240 MPa: blue). The uncertainties were calculated from the repeatability of the measured sizes, which were obtained from three separate measurements. The dotted curves were calculated using Equations (4)–(6), respectively.

**Figure 3:**
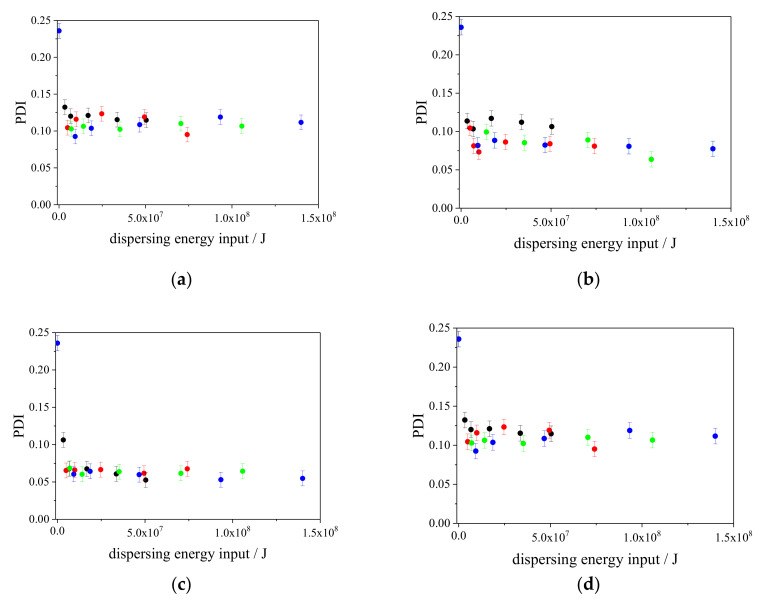
Plots of the PDIs of CaCO_3_ particles in various aqueous media as functions of the dispersing energy input, as determined by DLS. The used surfactants are (**a**) Triton X-100, (**b**) SDS, (**c**) Softanol-70, and (**d**) CTAB. The plots are for different pressures in the narrow channel (80 MPa: black, 120 MPa: red, 180 MPa: green, and 240 MPa: blue). The uncertainties were calculated from the repeatability of the observed sizes, which were obtained from three separate measurements.

Using the wet-jet milling system, the particles were dispersed as small aggregates/agglomerates or constituent particles. In this study, DLS assessments were performed for all of the aqueous CaCO_3_ particle suspensions to obtain the ensemble particle size in the liquid phase. Although simple assumptions were used (e.g., a monomodal size distribution and a spherical shape) in the DLS characterization of the CaCO_3_ particles in the aqueous suspensions, the DLS method is helpful for examining the differences in the hydrodynamic sizes of the various aqueous CaCO_3_ particle suspensions. We applied the cumulant method for the auto correlation function data analysis because the fitting data by cumulant method agreed with the raw photon correlation function. The uncertainties of the measured diameters were calculated from the repeatability of the observed values, which were obtained from at least three separate measurements. During the wet-jet milling dispersing process, we performed particle size characterization by DLS to monitor the change in the size as a function of the number of passes. In this study, we monitored the sizes of at least five different number of passes from 0 to 30 passes in one dispersing shear force condition. In addition, we used four dispersing shear forces (80 MPa, 120 MPa, 180 MPa, and 240 MPa), therefore, we obtained at least 20 different energy input conditions for one sample. Four different samples were examined under these different conditions. The results are plotted in Figure 2a–d. Additionally, the polydispersity indices (PDIs) at different times are shown in Figure 3a–d. All of the figures are plotted as functions of the dispersing energy input.

According to the DLS analysis, the mean particle sizes of the CaCO_3_ particles dispersed by wet-jet milling became clearly smaller with the increase in the dispersing energy input values, except for the samples using CTAB as the surfactant. The relationship between the mean particle size of the CaCO_3_ particles and the dispersing energy input value was unrelated to the pressure in the narrow channel from 80 to 240 MPa. To the best of our knowledge, studies using the wet-jet milling method have used the pressure in the narrow channel and the number of passes (dispersing passes in the narrow channel) as the main parameters to describe the condition of the dispersing particles. However, a serious problem is that the dispersing tendencies of the particles using the wet-jet milling method are not simply represented as functions of pressure. Thus, even under the same experimental conditions, such as the pressure in the narrow channel and the number of passes, wet milling users cannot reproduce their experimental results. Therefore, the finding of the close relationship between the particle size and dispersing energy input value is quite significant because the condition of dispersing particles using the wet-jet milling method is represented/evaluated by just one parameter. In addition, the most significant impact of this finding on the process of dispersing CaCO_3_ particles using the wet-jet milling method is the easy estimation of the appropriate condition of the dispersing process using wet-jet milling. This will enable us to control the required mean size of CaCO3 particles based on the idea of dispersing energy input. In Figure 2a–c, the relationships between the mean particle size and the dispersing energy input for different surfactants are represented by Equations (4)–(6), respectively.

(4) $$d=139.71+31.68\times e^{\left(-\frac{E}{43.01\times 10^{4}}\right)}+130.27\times e^{\left(-\frac{E}{91.33\times 10^{6}}\right)}$$

(5) $$d=115.38+45.15\times e^{\left(-\frac{E}{51.51\times 10^{6}}\right)}+148.05\times e^{\left(-\frac{E}{17.79\times 10^{7}}\right)}$$

(6) $$d=164.88+63.05\times e^{\left(-\frac{E}{23.44\times 10^{5}}\right)}+54.25\times e^{\left(-\frac{E}{80.20\times 10^{6}}\right)}$$

In the equations, d is the estimated mean size (nm), and E is the dispersing energy input (J). For example, using Equation (6), it is predicted that the mean size of the CaCO_3_ particles in the Softanol-70 aqueous phase can be controlled at approximately 200 nm at a dispersing energy input of 3.5 × 10^7^ J. Based on Figure 1, the dispersing condition to produce approximately 200 nm CaCO_3_ particles in the Softanol-70 aqueous phase is accomplished with twenty passes (80 MPa), fifteen passes (120 MPa), ten passes (180 MPa), and five passes (240 MPa). This finding solves the difficulty in predicting/optimizing the dispersing condition in the wet-jet milling method.

As shown in Figure 2a–c, the size reduction tendencies were different depending on the type of the surfactant. This indicates that the best surfactant for dispersing CaCO_3_ particles in an aqueous phase is Softanol-70 because the weak dispersing energy input induces smaller sizes of CaCO_3_ particles in the aqueous phase compared with those in the other two surfactants (Triton X-100 and SDS). As shown in Figure 3a–c, the values of the PDI also provide important information. The PDI value of the CaCO_3_ particles dispersed using Softanol-70 was smaller than those when they were dispersed using the other two surfactants (Triton X-100 and SDS) at the same dispersing energy input. This indicates that the best surfactant for dispersing CaCO_3_ particles in an aqueous phase is Softanol-70. Comparing Figure 3a,b, at the same dispersing energy input, the PDI values in Figure 3b are slightly smaller than those in Figure 3a. These results indicate that SDS is a more effective surfactant in dispersing of CaCO_3_ particles in an aqueous phase than Triton X-100 is. This is because SDS induces a narrower size distribution of CaCO_3_ particles in the aqueous phase than Triton X-100 does, even though the mean sizes of CaCO_3_ particles at the same dispersing energy input are similar.

As shown in Figure 2d, the CaCO_3_ particles dispersed by wet-jet milling using CTAB as the surfactant were smaller for a weak dispersing energy input compared with those using Triton X-100 and SDS. For example, at a dispersing energy input of 1.0 × 10^7^ J, the CaCO_3_ particles were approximately 250 nm in size when they were dispersed using Triton X-100 and SDS, whereas the size was approximately 200 nm when they were dispersed using CTAB. Interestingly, in the case of CaCO_3_ particles dispersed using CTAB, the CaCO_3_ particles at such a weak dispersing energy input (e.g., 1.0 × 10^7^ J) were similar in size to those that were dispersed when Softanol-70 was used as the surfactant (approximately 200 nm). This is despite the PDI values of the CaCO_3_ particles dispersed by CTAB being larger than those of the CaCO_3_ particles dispersed by Softanol-70, as shown in Figure 3c,d. These phenomena indicate that CTAB endows a higher dispersibility to CaCO_3_ particles in an aqueous phase than Triton X-100 and SDS do. Although the mean particle size of the CaCO_3_ particles dispersed using CTAB was close to that which was achieved using Softanol-70 at a relatively weak dispersing energy input, Softanol-70 has a higher dispersing capability for CaCO_3_ particles in an aqueous phase than CTAB does. This is because the size distribution of particles dispersed by Softanol-70 is clearly narrower than that of the particles dispersed by CTAB, as shown in Figure 3c,d.

However, at a relatively strong dispersing energy input, the mean sizes of the CaCO_3_ particles using CTAB as the surfactant increased with the increase in the dispersing energy input, as shown in Figure 2d. This tendency is completely different from those of the CaCO_3_ particles produced using the other surfactants. In this study, we determined that the size enlargement of the CaCO_3_ particles at a stronger dispersing energy input in the case of CaCO_3_ particles dispersed using CTAB may be caused by the change in the surfactant micelles. Therefore, to assess the change in the surfactant micelles, we used PFG-NMR.

### 3.3. PFG-NMR Characterization of the Micellar Sizes of Various Surfactants in the Aqueous CaCO_3_ Particle Suspensions

**Figure 4:**
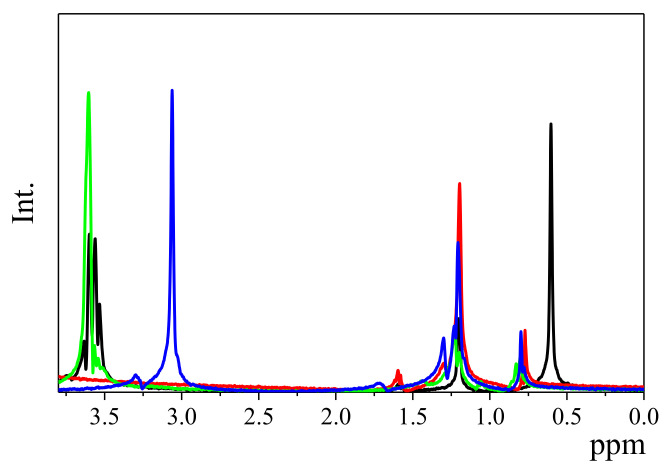
^1^H NMR spectra of various surfactants in aqueous CaCO_3_ particle suspensions. Triton X-100 (black), SDS (red), Softanol-70 (green), and CTAB (blue) were used as surfactants. All of the spectra were obtained using the PFGSTE pulse sequence at a gradient strength of 65 G cm^−1^.

Figure 4 shows the ^1^H spectra of the respective surfactants in the CaCO_3_ particle suspensions measured using the PFGSTE pulse sequence at a gradient strength of 65 G cm^−1^. The peak at 3.6 ppm was assigned to the methoxy group, the carbonyl peaks appeared at 3.0–2.5 ppm, and other alkyl protons appeared at 1.5–0.5 ppm, such as CH_2_CH_3_ and CH_2_CH_2_. Because the peaks at these chemical shifts for all of the surfactant molecules were sufficiently strong enough for the PFG-NMR measurements to be performed, we utilized these peaks for characterizations.

**Figure 5:**
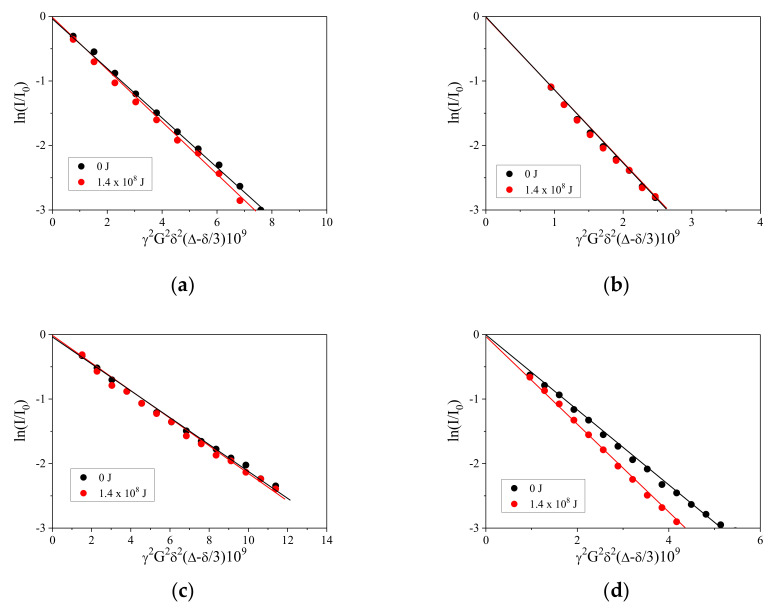
Examples of PFG-NMR spin-echo signal attenuation plots of respective surfactants in aqueous CaCO_3_ particle suspensions: (**a**) Triton X-100, (**b**) SDS, (**c**) Softanol-70, and (**d**) CTAB at δ = 1 ms for diffusion time of Δ = 50 ms. The solid line represents linear regression. Black circles and lines are for the samples before wet-jet milling. Red colored circles and lines are for the samples dispersed at the highest dispersing energy input (approximately 1.4 × 10^7^ J). Based on the Stokes–Einstein assumption, the estimated micellar size of the surfactants is approximately 1–8 nm.

**Figure 6:**
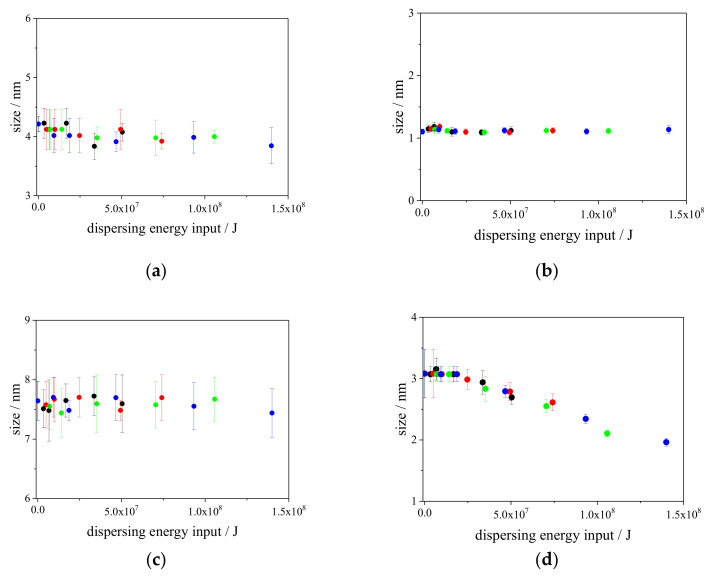
Plots of the micellar sizes of various surfactants in aqueous media as functions of the dispersing energy input, as determined by PFG-NMR. (**a**) Triton X-100, (**b**) SDS, (**c**) Softanol-70, and (**d**) CTAB were used as surfactants. The plots are for different pressures in the narrow channel (80 MPa: black, 120 MPa: red, 180 MPa: green, and 240 MPa: blue). The uncertainties of the diffusion coefficients were calculated according to [29].

The plots of the PFGSTE echo signal attenuation for the aqueous CaCO_3_ particle suspensions using various surfactants are shown in Figure 5. The attenuation plots are approximately linear, and they do not rely on the diffusion time, suggesting that the industrial surfactants follow a monomodal size distribution. The diffusion coefficients of all of the surfactant molecules before wet-jet milling which were calculated by linear regression (the solid lines in Figure 5) were 1.11 ± 0.49 × 10^−10^, 4.28 ± 0.22 × 10^−10^, 6.50 ± 0.33 × 10^−11^, and 1.60 ± 0.06 × 10^−10^ m^2^ s^−1^ for Triton X-100, SDS, softanol-70, and CTAB, respectively. The uncertainties of the diffusion coefficients were calculated on the basis of [29]. Based on the Stokes–Einstein assumption, the calculated micellar surfactant sizes were approximately 1–8 nm, indicating the surfactants were not solved as individual molecules, and instead they took the micellar structures. However, at the highest dispersing energy input (approximately 1.4 x 10^7^ J) in this study, the calculated diffusion coefficients of all of the surfactant molecules were 1.28 ± 0.15 × 10^−10^, 4.39 ± 0.16 × 10^−10^, 6.59 ± 0.35 × 10^−11^, and 2.69 ± 0.16 × 10^−10^ m^2^ s^−1^ for Triton X-100, SDS, Softanol-70, and CTAB, respectively. As shown in Figure 5, the attenuation plots of the different surfactant micelles present two different trends. For the first type, the slopes of the attenuation plots increase with an increasing dispersing energy input, suggesting that the size of the surfactant micelles decreased with an increasing dispersing energy input, based on the Stejskal–Tanner diffusion equation (Equation (2)). This tendency can be seen in Figure 6a,d (TritonX-100 and CTAB, respectively), however, the degree by which the slopes increased differ relying on the type of surfactant. Specifically, the slope at the highest dispersing energy input (approximately 1.4 × 10^7^ J) in CTAB is changed significantly from the initial value, whereas this change in Triton X-100 is small. For the second type, it is observed that there is approximately no change in the slope at the highest dispersing energy input (approximately 1.4 × 10^7^ J) from the initial slope. In contrast to the first tendency, this observation indicates that there were approximately no changes in the diffusion coefficients of the surfactant micelles on dispersing using the wet-jet milling method. This trend can be seen in Figure 6b,c (for SDS and Softanol-70, respectively).

To visualize the changes in the micellar sizes of the surfactants in each aqueous CaCO_3_ particle suspension, the sizes were calculated from the observed diffusion coefficients using the Stokes–Einstein equation (Equation (1)). The calculated changes in the micellar sizes of the surfactants as a function of the dispersing energy input are plotted in Figure 6. As shown in Figure 6d, the change in the size of CTAB micelles can be clearly observed, whereas those in the micellar sizes of the surfactants in aqueous CaCO_3_ particles suspension cannot be found in Figure 6a–c. This occurred even though the micellar sizes of Triton X-100 slightly decreased with the increase in the dispersing energy input. After adding the highest dispersing energy input (approximately 1.4 × 10^7^ J), the micellar sizes of the two surfactants did not change (SDS: 1.1 nm and Softanol-70: 7.5 nm), whereas those of the other two surfactants changed (Triton X-100 (4.3 nm to 3.9 nm) and CTAB (3.1 nm to 1.9 nm)). Interestingly, the tendencies of the changes in the micellar sizes were estimated to be related to the changes in the size of CaCO_3_ particles in the aqueous medium. Specifically, after adding the dispersing energy input by wet-jet milling, the mean sizes of CaCO_3_ particles reduced with the increase in the dispersing energy input when the sizes of the micellar sizes of the surfactants were not changed or slightly changed. Concurrently, in the case of a large change in the micellar sizes of the surfactant, such as with CTAB, the reduction in the mean sizes of the CaCO_3_ particles was disturbed, and then, the size of the CaCO_3_ particles increased with increasing dispersing energy input. Comparing Figure 2d and Figure 6d, the CaCO_3_ particles increased in size when the relative reduction in the micellar sizes of CTAB was at approximately 20 % (at approximately 5.0 × 10^7^ J). In the case of Triton X-100, the reduction in the micellar sizes was approximately 10 % at the highest dispersing energy input in this study, indicating this degree of the reduction of the micellar sizes is not critical in maintaining the stability of the sizes of CaCO_3_ particles in aqueous media. These results suggest that wet-jet milling affects the sizes of CaCO_3_ particles and some types of surfactant micelles, resulting in an adverse increase in the size of the CaCO_3_ particles.

### 3.4. DLVO Theoretical Assessment for Aqueous CaCO_3_ Particle Suspensions

To explain our observation related to the dispersibility of CaCO_3_ particles suspension under various dispersing conditions, we performed the measurements of the zeta potentials for all of the samples. The observed zeta potentials of the CaCO_3_ particles were −16.3 ± 0.5 (with Triton X-100 as the surfactant), −27.2 ± 0.6 mV (with SDS as the surfactant), −19.1 ± 0.7 mV (with Softanol-70 as the surfactant), and −42.8 ± 1.0 mV (with CTAB as the surfactant). The zeta potentials of aqueous CaCO_3_ particle suspensions without any surfactant were also measured. The repeatability of the observed zeta potentials was used as uncertainties in this study, which were obtained from at least three separate zeta potential measurements. Depending on the type of surfactant molecules, the observed zeta potentials of CaCO_3_ particles differed.

According to the Deryaguin–Landau–Verwey–Overbeek (DLVO) theory [30,31], two types of interactions are attributed to the dispersibility of CaCO_3_ particle dispersion, such as van der Waals attractive interactions and electrostatic repulsive interactions. With regard to the electrostatic repulsive energy, it is not equal to the zeta potential. However, the zeta potential is considered to be the potential for the slip face where a liquid starts to flow inside the electrical double layer formed on the hydrodynamic surface of a material. Therefore, the zeta potential is a significant factor for the evaluation of the electrostatic repulsive interaction between the CaCO_3_ particles in a liquid medium.

In the DLVO theory, the inter-particle van der Waals energy can be represented as follows:

(7) $$V\left(A\right)=-\frac{A}{6}\left(\frac{2}{4B+B^{2}}+\frac{2}{{\left(2+B\right)}^{2}}+ln\left(\frac{4B+B^{2}}{{\left(2+B\right)}^{2}}\right)\right)$$

where *A* is the Hamaker constant, *B* = 2*r*/*d*, *d* is the particle size, and *r* is the distance between the interacting two particles. By contrast, the electrostatic repulsive interaction between the CaCO_3_ particles and surfactant micelles are represented as follows:

(8) $$V\left(R\right)=\pi \varepsilon_{0}\varepsilon d\Psi^{2}e^{\kappa r}$$

and

(9) $$\frac{1}{\kappa }=\sqrt{\frac{\sum_{i}\rho_{\infty i}e^{2}z_{i}^{2}}{\varepsilon_{0}\varepsilon k_{B}T}},$$

where *ε*_0_ is the permittivity of vacuum, *ε* is the dielectric constant of the electrolyte solution, *e* is the elemental charge, *k_B_* is the Boltzmann constant, *T* is the absolute temperature, *ρ*_∝i_ is the ion number density in the bulk electrolyte, *z* is the valence of ion *i*, and *κ* is the inverse of the Debye length. Considering the two interaction energies, the total DLVO force, *V_total_*, is given as follows:

(10) $$V_{total}=V\left(A\right)+V\left(R\right)$$

In the DLVO theory, *V*(*A*) is attributed to the attractive energy between two particles if their sizes are the same. As shown in Figure 2d, the re-agglomeration of CaCO_3_ particles in the CTAB aqueous solution starts at a dispersing energy input of approximately 7.5 × 10^7^ J, and the produced agglomerates never re-disperse after constituent dispersing by wet-jet milling (with a much higher dispersing energy input). The increase in the size decreases the attractive interaction energy according to Equation (7), however, redispersion never occurs, suggesting that the main factor for this dispersing system is not equal to the van der Waals attractive energies. By contrast, when we assume that the zeta potentials are equal to the surface potentials of the CaCO_3_ particles, as shown in Equation (8), the electrostatic repulsive energies of the CaCO_3_ particles using the various industrial surfactants as dispersant are different because of the difference in the values of the observed zeta potentials (from −16 mV to −43 mV). Specifically, according to the observation of the zeta potential, the repulsive energy of the CaCO_3_ particles in the aqueous CTAB solution was higher compared with those in the other surfactant aqueous solutions. Nevertheless, the reaggregation of CaCO_3_ particles occurred in the aqueous CTAB solution, as shown in Figure 2d, whereas no size changes were observed for the CaCO_3_ particles in the other surfactant solutions. In this case, Figure 6 suggests that the surfactant micellar sizes have an important role to induce the sufficient dispersibility of CaCO_3_ particles in aqueous media. For the CaCO_3_ particles in the aqueous CTAB solution, the surfactant micelle sizes were reduced with the increase in the dispersing energy input, indicating that the electrostatic repulsive energies induced by the surfactant micelles decreased with a decrease in the sizes of the surfactant micelles, as expressed in Equation (8). By contrast, the sizes of the two surfactant micelles (SDS and Softanol-70) did not change by wet-jet milling at any dispersing energy input, indicating that the electrostatic repulsive energies caused by these surfactant micelles did not change in their dispersion systems. Although the sizes of the micelles for Triton X-100 slightly changed, the size changes are estimated to be critical to reduce the dispersibility of the CaCO_3_ particles in aqueous media. This is because the size change of the micelles of CTAB was 40 % at an energy input of approximately 1.4 × 10^7^ J, whereas that of Triton X-100 was 10 % at the same energy input. Relatively small changes in the micelle sizes might not affect the dispersibility of the CaCO_3_ particles in aqueous media. According to the DLVO theory, irregular changes in the dispersibility of CaCO_3_ particles in the aqueous CTAB solution can therefore be clearly explained by the change in the micellar sizes of CTAB. We previously observed similar phenomena (change in the micelle sizes of the surfactant affected by the dispersibility of particles in liquid media) in the case of dispersing carbon black particles using ultrasonication [10]. Thus, similarly, the micellar sizes of surfactants are crucial for inducing and maintaining the dispersibility of CaCO_3_ particles in aqueous media.

## 4. Conclusions

In this study, we established a new concept—dispersing energy input—in the wet-jet milling method to disperse materials in a liquid phase. The wet-jet milling method has been used in the preparation of soft material suspensions. However, the shear forces are the main driving force of dispersing, and they are weaker than they are in other dispersing methods, such as the ultrasonication dispersing method. Therefore, the control parameters of the wet-jet milling method for dispersing are the pressure of the narrow channel that the target liquid including particles is passed through and the number of passes. The above indicates that the former one is not a universal parameter to setup the dispersing condition by various wet-jet milling users because the values of this pressure are dependent on the size (diameter and length) of the narrow channel. A simple optimization/prediction method of dispersing desired sizes of target particles by the wet-jet milling method is desired. Using the idea of the dispersing energy input, it was found that the dispersing size of particles can be easily optimized/predicted. We demonstrated the usability of the concept by preparing CaCO_3_ particles in aqueous suspensions with various surfactants using the wet-jet milling method as an example. For instance, it was easy to estimate the dispersing condition to produce approximately 200 nm CaCO_3_ particles in the Softanol-70 aqueous phase, and this was accomplished with 20 passes (80 MPa), 15 passes (120 MPa), 10 passes (180 MPa), and 5 passes (240 MPa) using the idea of energy input. This finding solves the difficulty in predicting/optimizing the dispersing condition in the wet-jet milling method. In addition, by conducting a PFG-NMR assessment, we also identified the important role played by the micellar sizes of surfactants in dispersing particles by wet-jet milling. The tendencies of the changes in the micellar sizes were estimated to be related to the changes in the size of the CaCO_3_ particles in the aqueous medium. It was found that with a large change in the micellar sizes of the surfactant, such as with CTAB, the reduction in the mean sizes of the CaCO_3_ particles is disturbed.

## Data Availability

Not applicable.
